# Identification of a novel CTCF mutation responsible for syndromic intellectual disability – a case report

**DOI:** 10.1186/s12881-017-0429-0

**Published:** 2017-06-15

**Authors:** Fatma Bastaki, Pratibha Nair, Madiha Mohamed, Ethar Mustafa Malik, Mustafa Helmi, Mahmoud Taleb Al-Ali, Abdul Rezzak Hamzeh

**Affiliations:** 1 Pediatric Department, Latifa Hospital, Dubai Health Authority, Dubai, United Arab Emirates; 2 Centre for Arab Genomic Studies, P.O. Box 22252, Dubai, United Arab Emirates

**Keywords:** CCCTC-binding factor, Intellectual disability, De novo mutation, Arab, case report

## Abstract

**Background:**

Autosomal dominant mental retardation 21 (MRD21) is a very rare condition, characterized by short stature, microcephaly, mild facial dysmorphisms and intellectual disability that ranged from mild to severe. MRD21 is caused by mutations in CCCTC-binding factor (CTCF) and this was established through only four unrelated cases, two of which had frameshift mutations. CTCF is a master transcriptional regulator that controls chromatin structure and may serve as insulator and transcriptional activator and repressor.

**Case presentation:**

This study presents, clinically and molecularly, an Emirati patient with de novo frameshift mutation in CTCF. This novel mutation was uncovered using whole exome sequencing and was confirmed by Sanger sequencing in the trio. In silico analysis, using SIFT Indel, indicates that this frameshift; p.Lys206Profs*13 is functionally damaging with the likely involvement of nonsense-mediated mRNA decay.

**Conclusions:**

Upon comparing the clinical picture of the herewith-reported individual with previously reported cases of MRD21, there seems to be many common symptoms, and few new ones that were not observed before. This helps to further define this rare condition and its molecular underpinnings.

## Background

The highly conserved CCCTC-binding factor (CTCF) is a remarkably versatile tool in eukaryotic cells, as it serves a myriad of functions in relation to epigenomic regulation of gene expression. This extensive regulatory role of CTCF is not limited to being an insulator that stops interactions between responsive promoters and nearby enhancers and silencers. CTCF is also involved in functional restructuring of chromatin, X chromosome inactivation and general genomic imprinting. Additionally, CTCF, along with cohesin, is involved in somatic recombination during lymphocyte diversification. Total loss of function of *CTCF* through homozygous deletion leads to early embryonic lethality in mice, which signifies the pivotal role of this regulatory factor in development [1].

The DNA-binding protein encoded by the *CTCF* gene contains eleven zinc finger (ZF) domains with many *CTCF* orthologs showing nearly complete homology. The versatility of CTCF stems from its ability to use different combinations of its zinc fingers to recognize a varied range of DNA sequences known as CTCF target sites (CTSes) [2]. *CTCF* has 12 exons, different combinations of which give rise to seven protein isoforms. The latter can be modified post-translationally by phosphorylation, sumoylation, and polyADP-ribosylation [3].

Given the abovementioned functions it is unsurprising that somatic *CTCF* mutations were linked to cancer (i.e. acute leukemia) and genomic CTCF/cohesin-binding sites were found to be major mutational hotspots in numerous malignancies [4]. Germline *CTCF* mutations are responsible for a specific phenotype in humans [5]; this phenotype is syndromic intellectual disability with microcephaly and growth retardation; the reported mutations are frameshift and missense mutations, which goes along with the results reported here. The aforementioned phenotype was named MRD21; autosomal dominant mental retardation 21 (MIM: 615,502), and its cardinal features include short stature, microcephaly, intellectual disability of various degrees and developmental delay. Patients also have minor facial dysmorphisms (e.g. small mouth, prominent incisors and thin upper lip) and may have heart anomalies, e.g. atrial septal defect (ASD) and patent ductus arteriosus (PDA) in addition to displaying an autistic behavior.

Here, we present the case of an Emirati female with a de novo frameshift mutation in exon 3 of *CTCF* that has never been reported before. This study describes the clinical features of the patient and sheds light on the underlying molecular defect using the few published cases of MRD21 and bioinformatic analysis of the functional consequences of the novel mutation.

## Case presentation

**Fig. 1:**
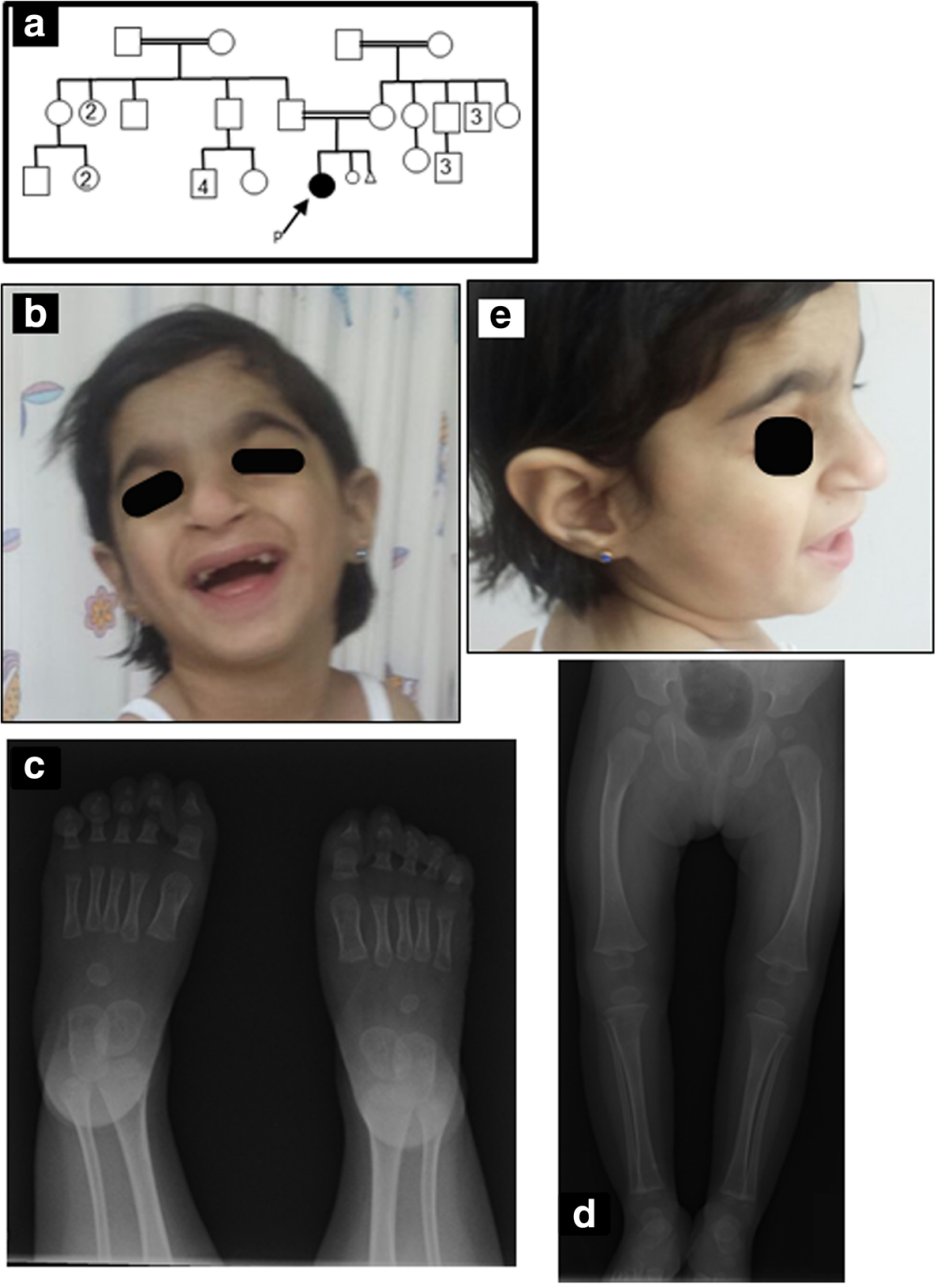
Panel (**a**) shows the pedigree chart of the patient. Panels (**b**) and (**e**) show clinical features of the *CTCF* mutation in the patient, which include numerous dysmorphisms such as microbrachycephaly, narrow forehead highly arched and bushy eyebrows, deep-seated eyes, broad nasal tip with wide everted nostrils, prominent incisors, and large ears with dysplastic helix and attached ear lobes. Panels (**c**) and (**d**) are X-ray radiographs showing the presence of diffuse generalized osteopenia

The patient was born to first cousin parents (Fig. 1a) at 26-weeks’ gestation due to fetal distress and intra-uterine growth retardation. Her birth weight at 0.56 Kg was under the 3rd centile, while head circumference (21 cm) was under the 10th centile. Parents’ heights and head circumferences are 154 cm and 56 cm for the mother, and 173 cm and 56 cm for the father, respectively. Although birth length of the patient could not be recalled, follow-up evaluations at 23 months and 3.5 years of age showed height to be at 70 cm and 82 cm, respectively, both below the 3rd centile. The patient stayed in the neonatal ICU for 5 months due to neonatal jaundice, recurrent chest infections, pulmonary hemorrhage, neonatal anemia, septicemia, chronic lung disease, GERD (Gastroesophageal reflux disease), and retinopathy of prematurity. She was seen at the genetic clinic for evaluation at 2-years of age (corrected age of 1 year and 8 months) because of failure to thrive, dysmorphic features, and developmental delay. She was found to be an alert, active and interactive girl, with height, weight and head circumference, all below the 3rd centile. She had distinct facial dysmorphic features, including microbrachycephaly, narrow forehead, mild synophrys, highly arched and thick eyebrows, long eye lashes, deep-seated eyes, microcornea, esotropia, midface retrusion, short nose, columella and philtrum, broad nasal tip with wide anteverted nares, cube shaped lips, prominent incisors, and large cupped ears with attached ear lobes (Fig. 1b and e). She had normal external female genitalia with hypoplastic labia majora. Partial syndactyly between the second and third toes was noted, as was bilateral clinodactyly of the little finger. Development was delayed, with the child being unable to roll over, reach, hold or transfer objects, wave, recognize parents, or sit for more than a few seconds, or walk more than a few steps, unsupported. Evaluation at 3.5 years confirmed the developmental and speech delay. By this time, she was unable to walk without support, and could only babble. She suffered repeated chest infections that necessitated her frequent hospitalization.

Echocardiography at birth had identified a small atrial septal defect secundum, and moderate PDA with volume overload. Subsequent repeating of ECG revealed a ligated PDA with mild mitral regurgitation, improving pulmonary hypertension, and a superior vena cava thrombus that was later resolved. Abdominal US revealed massive gastro-esophageal reflux. Brain US was normal at 5 months of age. Skeletal survey showed a diffuse osteopenic texture of the examined bones along with a brachycephalic type of craniosynostosis (Fig. 1c and d). She had 11 pairs of ribs.

A detailed study of the family history did not show any relatives with a similar presentation. The mother had two miscarriages after the proband’s birth.

### Molecular results

Full informed consent was obtained from the guardian of the individual participant for whom identifying information is included in this article. Then, peripheral blood samples were collected from the patient and her parents. Thereafter, DNA was extracted from blood samples according to standard protocols. Amplicon library construction for whole exome sequencing, exome capture, sequencing, and standard data analysis were performed by Sengenics (Kuala Lumpur, Malaysia). SureSelect Human All Exon V5 exome capture kit (Agilent Technologies) was used for library preparation and the samples are being sequenced at a minimum of 100X mean standard coverage using Illumina HiSeq 2500 platform. The number of genes covered is 21,522 and 98.57% of targets had a coverage of at least 20×. A total of 102,391,432 sequence reads was obtained and 96,052,057 of them (99%) were mappable, while the resulting number of variants was 103,118. These variants were filtered according to quality, frequency, genomic position, consequences on encoded proteins, pathogenicity and previously reported associations with the phenotype. Of particular interest were the 49,475 homozygous variants found (parents are 1st degree cousins), multiple filtration steps were applied considering functional consequences and minor allele frequency (MAF) as well as any overlaps with genes that were previously associated with at least one of the symptoms described in the patient; i.e. a restricted gene list was used based on the clinical presentation of the patient.

Findings from WES were confirmed by Sanger sequencing in the trio. Reference sequence identifiers for wild-type *CTCF* and *SETD5* were obtained from GenBank; NCBI Reference Sequences: NM_006565.3 and NM_001080517, respectively. The functional consequences of the variant were obtained using SIFT Indel [6], which is available at http://sift.bii.a-star.edu.sg/www/SIFT_indels2.html. The latter algorithm predicts the effects of indels and it is an extension of the SIFT (Sorting Intolerant From Tolerant) algorithm, which predicts the effect of amino acid substitutions [7]. The query phrase used was “16,67,645,346,67,645,350,1,AAAG”, which contained the chromosomal coordinates of the indel, as per the genome assembly GRCh37.

**Fig. 2:**
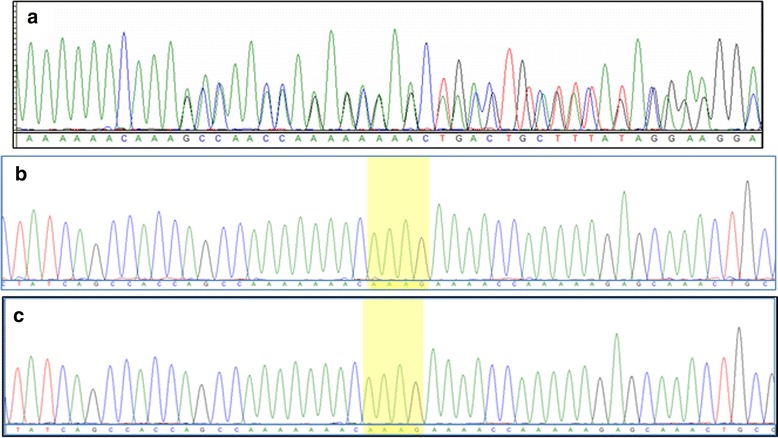
Sequence chromatograms showing the novel *CTCF* frameshift mutation in a heterozygous state in the patient; Panel (**a**). Both parents were found to harbor wild type *CTCF*; Panels (**b**) and (**c**), in which the AAAG that is deleted by the mutation is highlighted

The patient has a normal karyotype and no abnormalities were detected using chromosomal microarray test. To uncover the molecular lesion that underlies the abovementioned phenotype, Whole Exome Sequencing (WES) was performed for the patient. WES revealed a heterozygous 4 bp-deletion in the *CTCF* gene at the following coordinates; at the genomic level “chr16: 67645346delAAAG” and the cDNA one “c.612delAAAG”. Upon sequencing *CTCF* in the parents, it was clear that the mutation appeared de novo in the child, as neither of parents harbored the mutation (Fig. 2). Using the SIFT Indel algorithm the effect of the deletion is “damaging”, as it results in a frameshift that is predicted to translate out of frame for stretch of 13 amino acids ending with a premature stop codon; p.Lys206Profs*13. This means that the resulting CTCF is truncated at the end of its N-terminal region and thus it lacks all its 11 zinc-finger domains and C-terminal region. The variant was not previously reported on any of the relevant databases such as dbSNP, ExAC, 1000-genomes and EVS. Additionally, the variant was not found in the GalaxC™ Allele Frequency Database which contains >2.5 million unique Middle Eastern pathogenic mutations and variants.

## Discussion and conclusions

The global regulatory role of CTCF in organizing chromatin structure explains the increasing number of studies that uncover its involvement in a wide range of human disorders. This involvement is not limited to malignancies but CTCF was also implicated in Huntington’s disease and autoimmune disorders [6–8]. Many studies have also highlighted the relationship between CTCF and fetal growth. For example, deletion of CTCF target sites in the *H19* differentially methylated region causes the overgrowth disorder Beckwith-Wiedemann syndrome, which is also associated with increased risk of childhood tumors [9, 10]. In fact, a strong association was detected between height and CTCF target sites on the genomic level, with a significant number of the latter was found to overlap with height-associated SNPs in humans [11]. Importantly, functional CTCF is required for experience-dependent gene regulation and, consequently , for memory formation and cognition in general, this makes for the strong connection between intellectual disability and *CTCF* mutations [12].

Expression studies in individuals with the *CTCF* frameshift mutations; c.375dupT and c.1186dupA, revealed nearly complete absence of the mutated alleles. This was explained by the role played by nonsense-mediated mRNA decay (NMD) in rendering the phenotype of total loss of function and haploinsufficiency [5]. Interestingly, nonsense-mediated mRNA decay was predicted by SIFT Indel algorithm, as the most likely outcome of the here-reported mutation (c.612delAAAG), and therefore it is possible that the same mechanism is underlying the phenotype of MRD21 in our patient. This is strongly supported by the pLI (Probability of LoF intolerance) score of 1.00 that is given to *CTCF* LoF mutations by ExAC database, as it signifies high intolerance to haploinsufficiency.

In addition to the two frameshift mutations, a missense one (c.1699C > T; p.Arg567Trp) was also reported by Gregor et al. 2013. This heterozygous mutation was deemed to perturb DNA binding affinity and specificity of CTCF through a dominant-negative effect or haploinsufficiency. Additionally, a forth case with intellectual disability harboring a heterozygous deletion that involved *CTCF* and seven other genes was reported. Therefore, the total number of reported cases that involve *CTCF* mutations is four, and all of them are de novo [5].

Many of the clinical features observed in our patient were also reported in the patients who were studied before [5], examples include stunted growth, microcephaly, developmental delay and facial dysmorphisms. The microcephaly in our patient was severe, with head circumference > 3 SD below the mean (40.3 cm at 3.5 years of age). Also, the patient in our study displayed certain symptoms that were not universally present in previously reported cases of MRD21. For instance, our patient suffered certain congenital heart defects such as atrial septal defect and patent ductus arteriosus as well as recurrent respiratory infections, and these were shared by individuals 1 and 2, from Gregor et al. 2013, respectively. Among the recurrent features were esotropia, many of the facial dysmorphisms and abnormalities of the fingers. However, the patient presented in this study has an additional feature affecting the skeletal system. The latter includes generalized low bone density and brachycephalic type of craniosynostosis. The causality of osteopenia is not necessarily linked to the here-reported *CTCF* mutation, as the patient harbors a heterozygous mutation in the gene *SH3PXD2B* (c.395C > G; p.Pro132Arg), which is predicted to be deleterious in silico. Homozygous mutations in *SH3PXD2B* cause Frank-ter Haar syndrome (FTHS), in which osteopenia was reported. Importantly, the mode of inheritance of FTHS is autosomal recessive, unlike MRD21; therefore, this symptom requires further investigations into its causality. Additionally, the patient has only 11 pairs of ribs. Importantly, WES revealed a certain heterozygous mutation (c.3221G > A; p.R1074Q) in *SETD5*, which is known to be involved in mental retardation type 23 (MIM: 615,761). However, sequencing the gene in the parents revealed that the mutation was inherited by the patient from her heterozygous father while the mother is wild type, which indicates that the latter variant is unlikely to be pathogenic. The patient suffered other health conditions that may have been essentially related to prematurity, but with studying more MRD21 cases from different ethnic backgrounds it will be easier to precisely define the clinical presentation of this condition.

In conclusion, we have introduced - molecularly and clinically - a novel variant in the *CTCF* gene in the context of autosomal dominant mental retardation 21. This is the first reported case of its kind from the Arab ethnicity and a step towards better and clearer identification of this very rare disorder.

## Abbreviations

ASD: Atrial septal defect
CTCF: CCCTC-binding factor
CTSes: CTCF target sites
ECG: Electrocardiogram
ExAC: Exome Aggregation Consortium
GERD: Gastroesophageal reflux disease
MAF: Minor allele frequency
MRD21: Autosomal dominant mental retardation 21
NMD: Nonsense-mediated decay
PDA: Patent ductus arteriosus
SIFT: Sorting Intolerant from Tolerant
SNP: Single nucleotide polymorphism
UAE: United Arab Emirates
VCF: Variant Call File
WES: Whole exome sequencing
ZF: Zinc finger
